# An Improvement to Conformer-Based Model for High-Accuracy Speech Feature Extraction and Learning

**DOI:** 10.3390/e24070866

**Published:** 2022-06-23

**Authors:** Mengzhuo Liu, Yangjie Wei

**Affiliations:** College of Computer Science and Engineering, Northeastern University, Wenhua Street 3, Shenyang 110819, China; 2071873@stu.neu.edu.cn

**Keywords:** end-to-end, Chinese speech recognition, capsule network, conformer, bi-transformer

## Abstract

Owing to the loss of effective information and incomplete feature extraction caused by the convolution and pooling operations in a convolution subsampling network, the accuracy and speed of current speech processing architectures based on the conformer model are influenced because the shallow features of speech signals are not completely extracted. To solve these problems, in this study, we researched a method that used a capsule network to improve the accuracy of feature extraction in a conformer-based model, and then, we proposed a new end-to-end model architecture for speech recognition. First, to improve the accuracy of speech feature extraction, a capsule network with a dynamic routing mechanism was introduced into the conformer model; thus, the structural information in speech was preserved, and it was input to the conformer blocks via sequestered vectors; the learning ability of the conformed-based model was significantly enhanced using dynamic weight updating. Second, a residual network was added to the capsule blocks, thus, the mapping ability of our model was improved and the training difficulty was reduced. Furthermore, the bi-transformer model was adopted in the decoding network to promote the consistency of the hypotheses in different directions through bidirectional modeling. Finally, the effectiveness and robustness of the proposed model were verified against different types of recognition models by performing multiple sets of experiments. The experimental results demonstrated that our speech recognition model achieved a lower word error rate without a language model because of the higher accuracy of speech feature extraction and learning using our model architecture with a capsule network. Furthermore, our model architecture benefited from the advantage of the capsule network and the conformer encoder, and also has potential for other speech-related applications.

## 1. Introduction

Accurate feature extraction from target speech signals is significant to speech processing tasks. As compared with traditional speech processing technologies, a deep learning framework exhibits the advantages of strong modeling capability, fast training speed, high learning accuracy, and high robustness. Therefore, in recent years, it has become one of the most commonly used methods in the speech processing field, such as speech enhancement [1], speech separation [2,3], speaker recognition [4], and speech recognition [5,6,7,8].

Earlier methods for speech feature extraction and learning under the framework of deep learning are mostly based on recurrent neural networks (RNNs), especially long short-term memory (LSTM) [9]. However, the limitations of RNNs, such as gradient vanishing and gradient exploding [10], influence the performance of these models based on RNNs. To address these problems, convolutional neural networks (CNNs) have been proposed [9,11]. However, there are also some limitations of CNN-based models in practical applications, including a large number of parameters, more data for learning, and less expressive ability. To improve the performance of CNNs, a series of new networks have been proposed: the AlexNet network first successfully applied many operations in CNNs, such as rectified linear units, dropout, and local response normalization, except for overlapping maximum pooling, novel data enhancement, and computing acceleration with a general processing unit [12]; VGGNet replaced the larger convolutional kernels in AlexNet with several consecutive 3 × 3 convolutional kernels and used 1 × 1 convolutional kernels for operations to improve the recognition performance of networks [13]; GoogLeNet realized high-speed computations using dense matrices [14]. These networks have improved the performance of CNNs from different aspects, however, their basic theory is still based on convolutional networks for exploration and optimization. Therefore, if they are used in speech-related tasks, there is a risk of losing valuable information in speech due to the maximum pooling operation in convolutional networks, thus, reducing the accuracy and speed of feature extraction and model learning.

**Figure 1:**
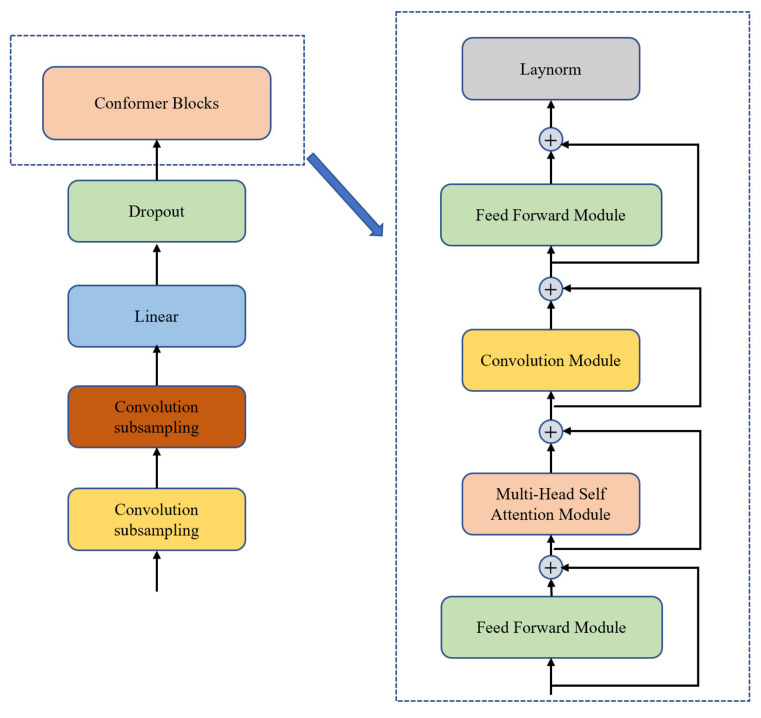
Conformer encoder model architecture [24].

As compared with CNNs, the basic theory of capsule networks is obviously different because they can reasonably preserve the detailed structure information throughout the network via sequestered vectors, rather than being lost and then recovered [15]. Owing to this advantage, they are widely used in image recognition [16], and recently, they have been introduced into speech emotion recognition [17]. Alternatively, benefiting from direct connection of arbitrary pairs of positions and easily parallelized computation, self-attention network has demonstrated promising results in a variety of natural language processing tasks [18,19,20]. Especially, the transformer model, which leverages multi-head attention and interleaves with feedforward layers, has been used for speech recognition with notable exceptions in recent years [21,22,23]. However, models with self-attention are less capable of extracting fine-grained local feature patterns. Therefore, the conformer encoder architecture, as shown in Figure 1, was proposed by Google Inc. to achieve very high-performance speech recognition, and is considered to be a convolution-augmented transformer [24].

As shown in Figure 1, in the conformer encoder model, a convolution subsampling network is commonly used to downsample or extract shallow features of speech signals for the conformer blocks. To improve the robustness in feature extraction that results from the local filtering and max-pooling techniques in the convolution network, a capsule network was introduced to replace the convolution subsampling network, and then, an end-to-end speech recognition model was proposed based on that architecture in this study. As compared with traditional speech recognition models, our proposed model is novel in several aspects and provides a new model architecture for speech feature extraction and learning. First, the convolution subsampling network in the original conformer encoder architecture is replaced by a capsule network. Thus, the structural information in speech can be preserved and input to the conformer blocks via sequestered vectors. Second, to improve the training speed of the capsule network, a layer of the residual network was added to deal with the degradation problem resulting from the increase in network layers. Finally, experiments with different architectures of end-to-end speech recognition models were conducted, and the results demonstrated that the proposed model architecture could obtain lower word error rates (WERs) with high efficiency. Furthermore, because our model architecture is able to improve the accuracy and speed of speech feature extraction and learning based on the conformer model, it also has potential for other speech-related applications.

The contents of this study are organized as follows: Firstly, in Section 2, the structure and information exchange equations in capsule networks are analyzed; secondly, a new end-to-end speech recognition model is proposed in Section 3; subsequently, in Section 4, the experimental results and error analysis based on different speech recognition models are given; finally, in Section 5, we provide the conclusions of this study.

## 2. Capsule Networks

**Figure 2:**
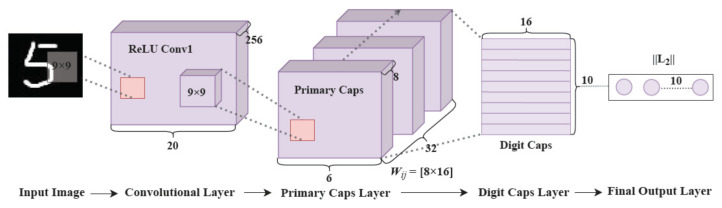
Architecture of the capsule networks.

Capsule networks are commonly used in image recognition because they can effectively solve problems in CNNs, such as indeterminate relationships between images and semantics and the lack of spatial layering or inference capability. The capsule network includes four parts: convolutional, primary capsule, digital capsule, and final output layers, as shown in Figure 2. The convolutional layer, as the starting point of the entire network, is mainly used to extract the low-level features of a target for preprocessing. The primary capsule layer contains the capsule network, which is mainly used to store the vectors of low-level features, accept the basic features detected by the convolutional layer, and generate the combination feature matrices, which are then sent to the digital capsule layer for the acquisition of high-level features.

The core of the digital capsule layer is the dynamic routing algorithm, which consists of four main steps:

(1) Multiply the input features by matrix ***W*** to realize the affine transformation,
  (1) $$\mu_{j|i}=W_{ij}\mu_{i}$$
  where ***μ****_i_* represents the input vector of the *i*-th capsule in layer *l*, ***W*** represents the relation weight matrix between layer *l* and layer (*l* + 1), and ***μ****_j_*_|*i*_ represents the vector of the *j*-th capsule in layer (*l* + 1) after affine transformation.
(2) Calculate the coupling coefficient vector ***c***,
  (2) $$c^{r}{}_{ij}=\frac{e^{b^{r}{}_{ij}}}{\sum_{k}e^{b^{r}{}_{ik}}}$$
  where ***c****^r^_i,j_* represents the coupling coefficient after the *r*-th iteration, *k* is the number of captures in layer (*l* + 1), ***b****^r^_i,j_* represents the aggregation result from the *i*-th capsule to the *j*-th capsule after the *r*-th iteration, and ***b***^0^*_i,j_* is zero.
(3) Calculate the weighted sum of the vectors after the *r*-th iteration,
  (3) $$s^{r}{}_{j}=\sum_{i}c^{r}{}_{ij}\mu_{j|i}$$
(4) Calculate the output vector of the *j*-th capsule in layer (*l* + 1) using the squash nonlinear activation function,
  (4) $$v^{r}{}_{j}=\frac{||s^{r}{}_{j}|{|}^{2}}{1+||s^{r}{}_{j}|{|}^{2}}\times \frac{s^{r}{}_{j}}{\left||s^{r}{}_{j}|\right|}$$

If the termination condition of the dynamic routing algorithm (normally the iteration number is set to three) is satisfied, the iteration stops and the feature extraction completes; ***v****_j_* is the output of the *j*-th capsule in layer (*l* + 1), and the magnitude of the output vector represents the probability of belonging to a class. Otherwise, the weight parameter ***b*** is updated and return to Step 2 for the next iteration,

(5) $$b^{r}{}_{ij}=b^{r}{}_{ij}+\mu_{j|i}v_{j}$$

From Figure 1, it can be observed that capsule networks have a shallow CNN layer and novel digital capsule and main capsule layers inside, so that the CNN layer can effectively extract the spatial and directional information of a speech signal based on the shallow features, such as the ability to obtain the location information of the pitch on the time and frequency axes, as well as the deep semantic logic information and the directional information of the bidirectional encoding [17]. Furthermore, capsule networks can use vectors to represent the features of speech signals, which can better classify the features and make parameter updating more accurate, therefore, they require less training data to achieve better training results as compared with other network structures [25]. However, current capsule networks have a small number of layers, the number of local parameters in each layer is very large, which easily causes the classification weights to be updated faster in the layers near the output and slower in the layers near the input in the back propagation, thus, the training speed of the entire network will be affected. Therefore, how to improve the training efficiency is a difficult problem in the applications of capsule networks. In the following section, we propose a method to solve this problem when we use the capsule network in speech recognition.

## 3. Improved Conformer-Based Model Architecture Based on the Capsule Network

**Figure 3:**
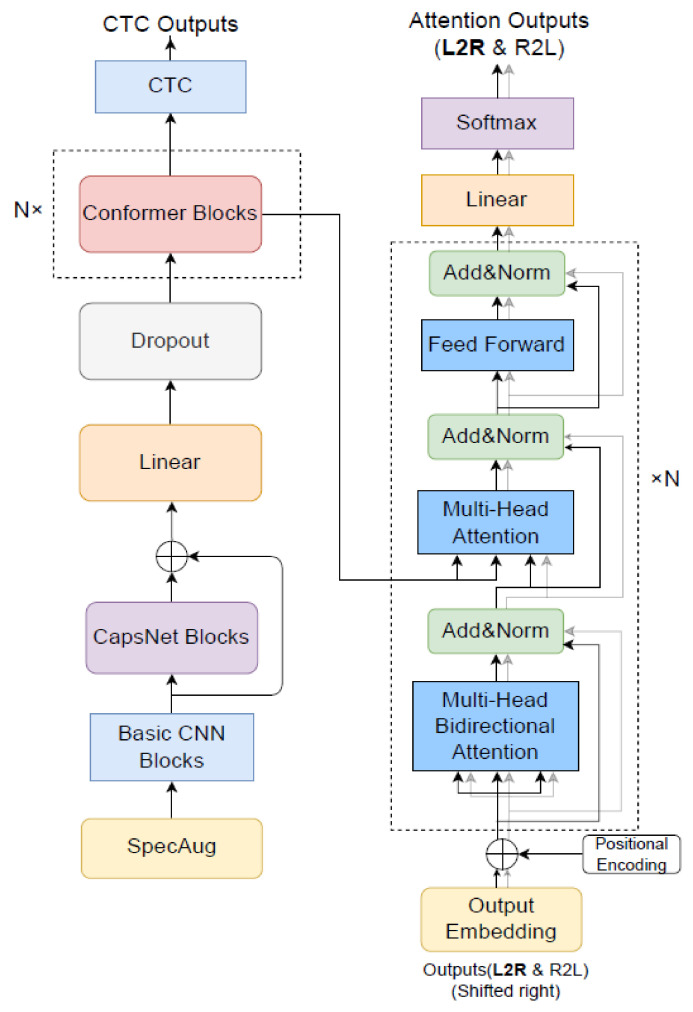
Architecture of our proposed speech recognition model.

In this study, the convolution subsampling network, shown in Figure 1, was replaced by a capsule network to downsample or extract shallow features of speech signals for the conformer blocks, and therefore, improve the accuracy of speech feature extraction. Then, based on this new model architecture that combined a capsule network and a conformer encoder, an end-to-end speech recognition model was proposed, which is shown in Figure 3. The model includes three core modules: the CapsNet blocks, conformer blocks, and bi-transformer decoder. The following describes our model according to its workflow.

### 3.1. Encoder

The speech features are extracted as input for our model. After data enhancement using SpecAugment [26], the shallow features are extracted by the basic CNN blocks as input to the CapsNet blocks in the capsule network. In this study, the CapsNet blocks consist of two internal layers: primary and digital capsule layers. The primary capsule layer spreads the speech data into multiple capsules, and then integrates them into a matrix, which is sent to the digital capsule layer for the acquisition of high-level features. The digital capsule layer follows the dynamic routing algorithm in Equations (1)–(5), and the magnitude of the output vector is the probability that the speech signal belongs to a category.

**Figure 4:**
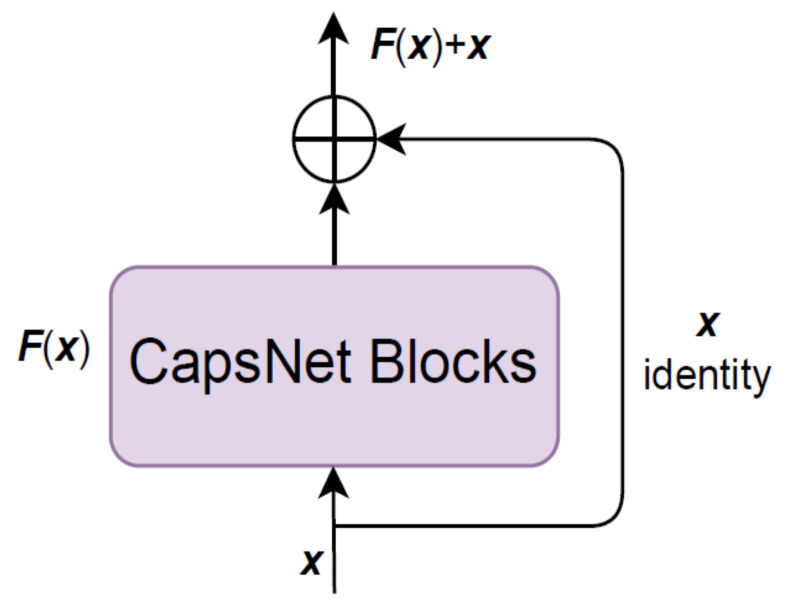
Combination of the capsule and residual networks.

First, to improve the training speed of the capsule network, we added a layer of residual network to the CapsNet blocks, as shown in Figure 4, because a residual network can deal with the degradation problem resulting from the increase in network layers [26]. After the introduction of a shortcut connection in the original network, our model allows data to flow across layers; thus, the initial learning goal of the network is changed, and the learning difficulty is reduced.

Assuming the input and output dimensions of the nonlinear unit in the neural network are the same, the function ***Z*** to be fitted within the neural network unit can be decomposed into two parts, given as:

(6) $$Z\left(x\right)=F\left(x\right)+x$$

where ***F*** represents the model function of the CapsNet blocks and ***x*** represents the system input.

Inside the network, the constant mapping ***Z***(***x***)→***x*** is learned, that is, the residual part ***F***(***x***) converges to 0. This is equivalent to replacing the learning target with ***Z***(***x***)-***x***, leading the whole structure to converge in the direction of iden tity mapping. This transformation can speed up the training of the capsule network and simplify the parameter optimization process. Then, ***Z*** is linearized to integrate the features and change the dimension, and the vector ***x*** is processed in the dropout layer to avoid over fitting:

(7) x=Dropout(Linear(Z))

**Figure 5:**
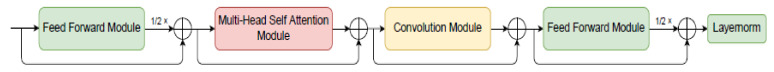
Diagram of the conformer blocks.

Finally, in this study, inputs, i.e., ***x***, to the conformer module enhance the performance of the encoder. Four modules are included in the conformer blocks: the feedforward, multi-head self-attention [27], convolution, and layer normalization modules; the specific structure is shown in Figure 5.

After feeding ***x*** into the conformer blocks, output ***y*** is fed through the feedforward module FFN, the multi-head self-attentive module MHSA, the convolutional module Conv, and the layer normalization module Layernorm. The specific process is as follows:

(8) $$x^{\prime }=x+\frac{1}{2}\mathrm{FFN}(x)$$

(9) $$x^{\prime \prime }=x^{\prime }+\mathrm{MHSA}(x^{\prime })$$

(10) $$x^{\prime \prime \prime }=x^{\prime \prime }+\mathrm{Conv}(x^{\prime \prime })$$

(11) $$y=\mathrm{Layernorm}(x^{\prime \prime \prime }+\frac{1}{2}\mathrm{FFN}(x^{\prime \prime \prime }))$$

### 3.2. Decoder

Because a bi-transformer model can give output as a left-to-right target sequence and a right-to-left target sequence with the advantage of better attention and more effective utilization of contextual information, our system uses the bi-transformer model as the decoder [28,29]. This decoder superimposes *N* sublayers into a multi-head attention model and feedforward network, and adds residual connections between the inputs and outputs of the sublayers. The bi-transformer decoder comprises two single weight-sharing decoders: one decodes from left to right to generate the forward context, and the other decodes from right to left to generate the backward context. The input information of the bi-transformer is obtained from the mapping at the output end of the encoder. Suppose that the output of the conformer is ***Y***, through matrix multiplication, it can be mapped into three matrices on different spaces: ***K***, ***Q***, and ***V***. Then, the forward and backward vectors are spliced to obtain a two-way dot product attention layer, and the attention scores ***H*** are calculated as:

(12) $$\vec{H}=\mathrm{Attention}\left(\vec{Q},\vec{K},\vec{V}\right),\overset{\leftarrow }{H}=\mathrm{Attention}\left(\overset{\leftarrow }{Q},\overset{\leftarrow }{K},\overset{\leftarrow }{V}\right)$$

Similarly, the multi-head attention value can be obtained by splicing:

(13) $$\mathrm{Multihead}\left(\left[\vec{Q};\overset{\leftarrow }{Q}],[\vec{K};\overset{\leftarrow }{K}],[\vec{V};\overset{\leftarrow }{V}\right]\right)=\mathrm{Concat}\left(\left[\vec{H_{1}};\overset{\leftarrow }{H_{1}}],\ldots ,[\vec{H_{h}};\overset{\leftarrow }{H_{h}}\right]\right)W^{O}$$

where ***W****^O^* is the trainable weight matrix, Concat is the concatenate operation, *h* is the number of heads.

Because the decoder is bidirectional, the decoding process is performed in both directions using the magnitude of the beam search value, and it stops when a termination symbol is predicted. The search process is as follows: At each time step, the alive hypothesis statement consists of two candidate statements guided by the special character *<L2R>*, where two candidate statements guided by the special character *<R2L>*, and the score of the completed sentence determines the best generated sequence. If the highest score is guided by the right-to-left sequence, the final predicted sequence must be reversed. This search method effectively solves the problem that the traditional transformer model can only decode in one direction, therefore, the consistency between the different directional hypotheses is improved. The loss function of the bi-transformer is defined as:

(14) $$L=\alpha \cdot L_{l2r}+(1-\alpha )\cdot L_{r2l}$$

where *α* is the inversion coefficient to balance the weight of the decoding order, and ***L****_l2r_* and ***L****_r2l_* represent the scores of the left-to-right and right-to-left decoding, respectively. In our system *α* = 0.5.

### 3.3. Decoding

**Figure 6:**
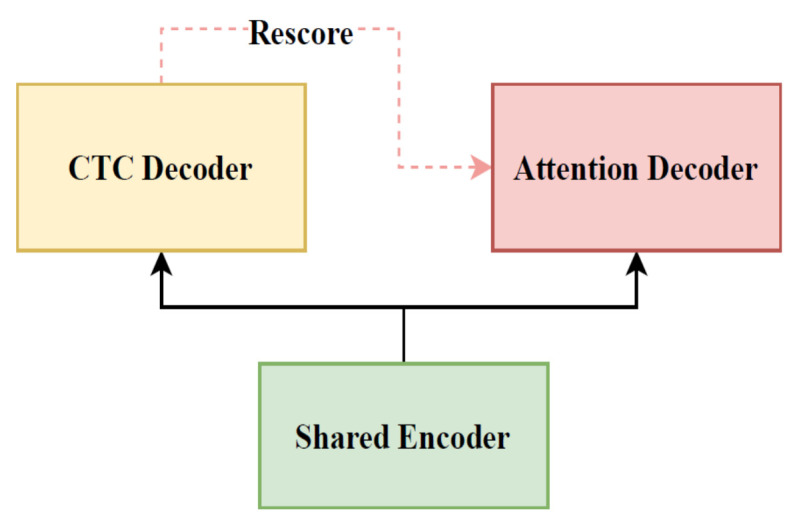
Structure of the U2 model.

**Figure 7:**
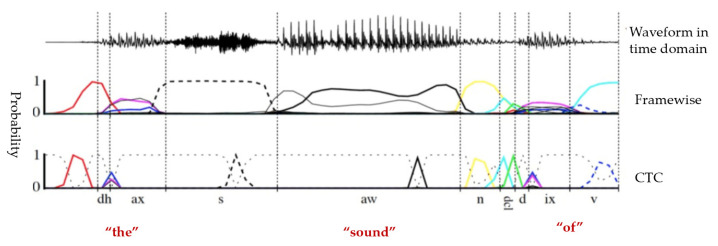
Framewise and CTC networks classifying a speech signal [31].

The purpose of this study was to design an offline speech recognition model architecture that does not contain a language model. Therefore, there is a high requirement for accuracy and stability, as well as trainability and decoding performance. The decoding framework in this study uses the U2 model with a two-channel joint connectionist temporal classification/attention-based encoder–decoder (CTC/AED) [30]; its structure is shown in Figure 6. The framework is derived from the traditional joint CTC-attention framework and includes three modules: CTC, attention, and shared encoders, where the connection between the CTC decoder and attention decoder is strengthened by the rescoring method. The CTC decoder consists of a linear layer that is used to transform the intermediate output of the encoder into the final output and complete the decoding using the attention rescoring method. Specifically, the CTC prefix beam search algorithm generates the *N*-best intermediate candidates, and then the attention decoder is used to jointly rescore these candidates with a shared encoder to obtain a more accurate final output, as shown in Figure 7. In addition, the U2 model uses the chunk training method to limit attention modeling. The principle is that the distribution of chunk sizes dynamically changes during the training process, and the attention model captures different information of various chunk sizes and learns how to make accurate predictions in different limited contexts to achieve a trade-off between decoding speed and accuracy.

The basic problem of speech recognition is to map a speech feature sequence to a word sequence, which can be solved by the Bayesian decision theory and probability function estimation theory [31,32]. First, the word sequence with the highest probability is estimated as follows:

(15) $$Z^{*}=\underset{Z\in V^{*}}{\arg\max}\left(p\left(Z|X\right)\right)$$

where Z* = {*z_n_* ∈ ***V***|*n* = 1, …, *N*} is the estimated word sequence with the highest probability in all the possible word sequences, ***X*** = {***x****_t_* ∈ **R***^D^*|*t* = 1, …, *T*} is the input speech feature sequence, ***x****_t_* is the *D* dimensional speech feature vector in the *t*-th frame, and *z_n_* the word at position *n* of the vocabulary ***V***.

According the Bayesian theory, *p*(***Z***|***X***) is transformed into:

(16) $$p\left(Z|X\right)=\frac{p\left(X|Z\right)*p\left(Z\right)}{p\left(X\right)}$$

Because in the decoding process, the input ***X*** does not change, *p*(***X***) is neglected. Therefore, Equation (16) is simplified as follows:

(17) $$Z^{*}=\underset{Z\in V^{*}}{\arg\max}\left(p\left(X|Z\right)*p\left(Z\right)\right)$$

where *p*(***Z***) is the probability of the output word sequence, which is described by the language model and *p*(***Z***|***X***) is the likelihood probability, characterized by the acoustic model.

Because the input audio frame is only about 20–30 ms and its information cannot cover a word or even a character, the general output modeling unit is a phoneme. The CTC method defines a path sequence ***C*** = {***c***_1_, …, *c_T_*}, where both the non-blank label and blank label exist, and the probability of the whole path sequence is composed of the label probability of each frame:

(18) $$p\left(C|X\right)=\prod_{t=1}^{T}y_{t}^{c_{t}}$$

where $y_{t}^{c_{t}}$ denotes the posterior probability of path *c_t_* in the *t*-th frame.

Define ***L*** as a label element table, the augmented letter sequence is defined with the blank symbol *b* as follows:

(19) $$L^{\prime }=L\cup \left\{\langle b\rangle \right\}$$

Define *Φ*: *L*^≤*T→*^*L*′ ^*T*^, and map the label sequence ***Z*** to the path sequence ***C***. Then, the probability of label ***Z*** is:

(20) $$p\left(Z|X\right)=\sum_{C\in \phi \left(Z\right)}p\left(C|X\right)$$

The factorization of the posterior probability *p*(***C***|***X***) is:

(21) $$p\left(C|X\right)=\sum_{z}p\left(C|Z,X\right)p\left(Z|X\right)\approx \sum_{z}p\left(C|Z\right)p\left(Z|X\right)$$

In the CTC method, *p*(***C***|***X***) is obtained using the conditional independence hypothesis, which can simplify the dependence of acoustic model and alphabetic model.

As compared with the CTC, the attention method estimates *p*(***C***|***X***) based on the rules of probability chain without any hypothesis [33] as follows:

(22) $$p\left(C|X\right)=\underset{≜p_{att}\left(C|X\right)}{\underbrace{\prod_{l=1}^{L}p\left(c_{l}|c_{1},\cdots ,c_{l-1},X\right)}}$$

where *p_att_*(***C***|***X***) is an object function based on attention, *p_att_*(*c_l_*|*c*_1_, …, *c_l_*_−1_, ***X***) is calculated as follows:

First, the input speech feature is encoded as:

(23) $$h_{t}=\mathrm{Encoder}\left(X\right)$$

The weight of attention is given as:

(24) $$a_{lt}=\left\{\begin{array}{l} Contentattetion\left(q_{t-1},h_{t}\right)\\ Locationattention\left({\left\{a_{t-1}\right\}}_{t-1}^{T},q_{l-1},h_{t}\right) \end{array}\right.$$

where *a_lt_* is the weight of attention, which denotes the soft connection *h_t_* of hidden vectors; Contentattention ( ) and Locationattention ( ) denote the content-based attention mechanism with and without convolution features, respectively.

For each output, the hidden vector of the lexical sequence is calculated as:

(25) $$r_{1}=\sum_{t=1}^{T}a_{tt}h_{t}$$

Then, the decoder network including a recurrent network based on the prior output *c_l_*_−1_ and hidden vector *q_l_*_−1_, except for the hidden vector *r*_1_, is used to calculate *p_t_*(*c_l_*|*c*_1_, …, *c_l_*_−1_, ***X***) as follows:

(26) $$p\left(c_{l}|c_{1},\cdots ,c_{l-1},X\right)=\mathrm{Decoder}\left(r_{l},q_{l-1},c_{l-1}\right)$$

In our training process, the CTC is combined with attention-based cross entropy [34,35], and a multi-objective learning framework is adopted to improve robustness. The loss function is denoted as:

(27) $$L\left(S\right)=\lambda L_{CTC}\left(S\right)+\left(1-\lambda \right)L_{AED}\left(S\right)$$

where ***L****_CTC_* and ***L****_AED_* are the loss functions of the CTC and attention decoders, respectively; *λ* is an adjustable parameter, which satisfies 0 ≤ *λ* ≤ 1; ***S*** denotes the training collection.

The ***L****_CTC_* is calculated as:

(28) $$L_{CTC}=-\ln\prod_{\left(X,Z^{*}\right)\in S}p\left(Z^{*}|X\right)$$

where Z* represents the correct label value, *p*(Z*|***X***) denotes the probability of obtaining the correct label corresponding to given ***X***, which can be calculated using the forward–backward algorithm as follows:

(29) $$p\left(Z^{*}|X\right)=\sum_{u=1}^{\left|y^{\prime }\right|}\frac{\alpha_{t}\left(u\right)\beta_{t}\left(u\right)}{q_{t}\left({c_{u}}^{\prime }\right)}$$

where ***c****′_u_* represents all the possible prefix paths ending with *u*, *α_t_*(*u*) represents the total probability of all possible prefix paths ending with the *u*-th label, *β_t_*(*u*) is the total probability of all possible suffixes starting from the *u*-th label, and *q_t_*(***c****′_u_*) represents the output value of ***c****′_u_* at time *t* after activation by the softmax function.

The ***L****_AED_* is calculated as:

(30) $$L_{AED}=-\sum_{u}\ln p\left(Z_{u}^{*}|X,Z_{1:u-1}^{*}\right)$$

where $Z_{1:u-1}^{*}$ represents the correct label before the current character *u*.

In summary, the flexible alignment of CTC accelerates the training process of the network, and the fusion of the CTC and attention decoder loss functions significantly simplifies the training channels and speeds up the model training process; therefore, training of the multiple branches of the encoder can be realized in speech recognition.

## 4. Experiment

### 4.1. Experimental Setup

To validate the model proposed in this study, a series of experiments were conducted on the open-source dataset AISHELL-1 which is an open-source Mandarin Chinese corpus comprising a 150-h training set, 10-h validation set, and a 5-h test set [36]. Furthermore, it has 4235 output units, including 4230 Chinese characters and five additional tokens [37]. In our experiments, the 80-dimensional Meier filter bank was used to compute the real-time acoustic features via Torchaudio, where the window length was 25 ms and the frame shift was 10 ms. In addition, two masks with the maximum frequency (*F* = 10) and maximum time (*T* = 50) were applied to effectively mitigate overfitting in SpecAugment. For the model parameters, we used a 12-layer conformer and transformer for encoding, where the feedforward position encoding dimension, model dimension, and number of attention heads were 2048, 256, and 4, respectively. The decoding side used a six-layer transformer and bi-transformer for decoding. For the optimizer, the Adam optimizer and the warmup strategy were introduced. The learning rate was set to 25,000, and the feedforward dropout was set to 0.1 to prevent overfitting. The batch size was set to 15, and the most suitable model was searched for through 150 rounds of training. In addition, to ensure the robustness of our model, the top five best models with a low loss in the development set during training were averaged to achieve the final model. The experimental system was Centos 7.9 (64 bits), the deep learning neural network framework training and testing software was Pytorch, and the GPU device model was Tesla T4 with a running memory of 15 GB.

To measure the accuracy of different models, the WER was used as an evaluation index, which can be expressed as:

(31) $$\mathrm{WER}=\left(S+D+I\right)/N\cdot 100\%$$

where *S* is character substitution, *D* is the deletion error, *I* is the insertion error, and *N* is the total number of words in the corresponding text of the original speech.

### 4.2. Performance Comparison and Ablation Analysis

#### 4.2.1. Comparisons of Different Encoders and Decoders

First, a series of cross-sectional comparison experiments were conducted to verify the accuracy of the proposed model. The models introduced for comparison included the following:

(1) Baseline models, i.e., compute library for deep neural network (CLDNN) with the structure of CNN × 2 + ResconvLSTM × 8 + DNN + CTC in [7], and LAS in [6];
(2) Models based on the capsule network combined with different encoders and decoders (Caps denotes the capsule network, C denotes the conformer, B denotes the bi-transformer, T denotes the transformer):, i.e., Caps-CB, Caps-CT, Caps-TT, and Caps-TB;
(3) Models with the original convolution subsampling network, i.e., CB, CT, TT, and TB.

In the experiments, the complete AISHELL-1 corpus was used for training, validation, and testing, and no pretrained model or language model was applied in the training. The WER and model parametric number were both calculated and compared, and the experimental results are shown in Table 1, from which the following conclusions can be obtained:

(1) Without the capsule network, the WER of the CB system that combined the conformer encoder and bidirectional transformer decoder was the lowest (6.21%). The WER of the TT system using only the transformer model was 7.5%, while the WER of the CT model using the conformer encoder and transformer decoder was 6.82%. That means, the recognition accuracy improved after the conformer model was used because of the encoder’s ability to incorporate multi-head attention global modeling and convolutional small-range feature extraction. Similarly, the WER of the TB system using the bidirectional transformer decoder was 6.55%, which was 0.95% lower than that of the TT system using the unidirectional transformer decoder. This is because the bidirectional transformer can fully use the contextual information, and decoding in both directions extends the modeling possibilities.
(2) After introducing the capsule and residual networks, the WER of the proposed Caps-CB model was the smallest (5.97%) under the same testing conditions; as compared with the models in [6] and [7], it decreased by 11.62% and 7.23%, respectively. The WERs of the Caps-TT, Caps-CT, and Caps-TB models were 7.31%, 6.36%, and 6.29%, respectively, which decreased by 0.19%, 0.46%, and 0.26%, respectively, as compared with the models without the capsule network. For our Caps-CB model, the WER decreased by 0.24% as compared with that of the CB model. This implies that the capsule network can enhance the feature extraction ability of these speech recognition models and can effectively extract deep features of speech signals, and also shows that the features in vector form are better than those in scalar form, which can enhance the performance of the encoder, and the capsule network can be well integrated with speech systems and applied to the field of speech recognition.
(3) For the number of parameters, without the capsule network, the number of parameters of the CB system was the largest, with a size of 50.0 M, whereas that of the TB model was the smallest, which was 33.8 M. Therefore, we can conclude that the addition of the encoder improved the recognition accuracy of the model, but increased the parameter number. As a result, the parameter number of these various models with the capsule network increased by approximately 2 M relative to the corresponding models without the capsule network. The ability to further improve the performance of the speech recognition system despite the small increase in the number of participants also demonstrates the power of the capsule network.

**Figure 8:**
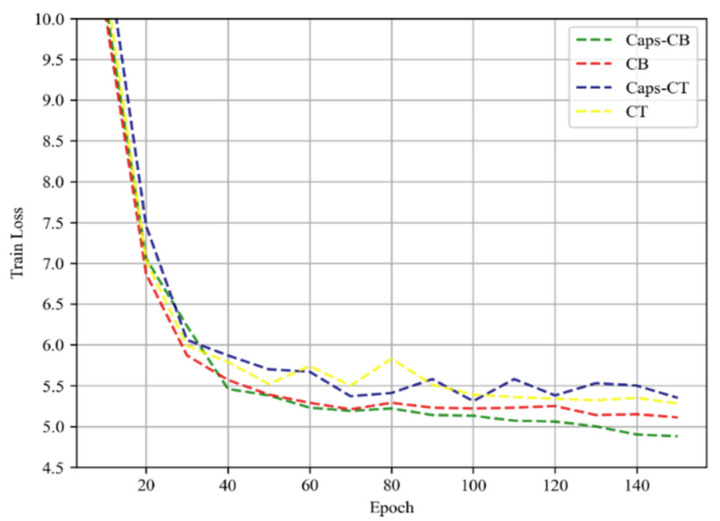
The loss function curves during training of different models.

Then, to observe the convergence state of model training, the loss function curves of the CT, Caps-CT, CB, and proposed Caps-CB models with an increasing number of iterations were drawn, and the results are shown in Figure 8, where the horizontal axis represents the number of iterations and the vertical axis represents the loss function value.

As can be observed from Figure 8, the loss function values of all models decrease significantly with an increase in training time, and the convergence speed of the Caps-CB model proposed in this study is significantly faster when the iteration number is more than 40. When the iteration number reaches 150, the loss function value of the Caps-CB model is approximately 50% of that of the Caps-CT model, followed by the CB model whose loss function value is approximately 75%. This implies that the combination of the conformer encoder and bidirectional transformer decoder can effectively improve the convergence speed, and after the subsampling network in the conformer is replaced by the capsule network, the convergence speed can be greatly increased.

#### 4.2.2. Combination with the Performer Encoder

It is well known that the training speed of the performer model is faster as compared with that of the conventional transformer [38], because it uses the FAVOR+ mechanism to construct an unbiased estimator for the attention mechanism matrix, resulting in a linear increase in resource requirements and a significant reduction in space and time complexity. In this experiment, we replaced the conformer encoder of our model with a performer, remaining the capsule network as the subsampling network, and the new model was called Caps-PB. Then, we tested the recognition performance and parameter quantity of the Caps-PB model and compared it with the PB, CB, and Caps-CB models. Table 2 presents the results.

From Table 2, we can observe that the WER of the Caps-PB model decreases by 0.23% as compared with the PB system, which means that enrollment of the capsule network can improve the recognition performance. The numbers of parameters of Caps-PB and PB are both lower than those of our model and CB because of the advantage of the performer; however, the WER of the Caps-PB model increases by 2.04% and 1.8% as compared with that of the Caps-CB and CB models, respectively. The reason for performance degradation of these models with the performer may be that the performer model is more effective for handling longer sequences (length > 5000) but less effective when dealing with shorter sequences. In the future, we plan to continue to explore new datasets that are more suitable for the Caps-PB model and to explore the performance of the capsule network on different datasets.

### 4.3. Analysis of Some Important Parameters

#### 4.3.1. Influence of Parameter Quantity

From Table 1, we can observe that the recognition WER of the Caps-CB model decreases by 0.24% relative to the CB model; however, the parameter quantity also increases by nearly 2 M. To explore whether the performance improvement was related to an increase in parameters, the intrinsic relationship between the number of parameters and performance was explored. First, we increased the number of parameters in the downsampling layer of the original CB model using layer superposition until it was as close as possible to that of the proposed Caps-CB model. Here, the enlarged CB model is referred to CB_enlarged. Then, we calculated the WERs of these models, and the experimental results are listed in Table 3. From this table, we can observe that the WER of the enlarged CB model is 0.03% lower than that of the original CB model, whereas it is still higher than that of our proposed Caps-CB model, although its parameter number increases by 1.95 M. These results show that the recognition ability of the convolutional network does not significantly improve after layer stacking, which does not change the working principle of the convolutional networks. This is also a good indication that the capsule network outperforms the CNNs correlation model with a novel change in terms of performance and is able to have good WER results with matching parameters.

#### 4.3.2. Influence of Router Iterations

In this experiment, the number of dynamic routing iterations in a capsule network determines the effect on parameter updating, even when it is set to three in most image recognition tasks using capsule networks; we tested the WER and the number of parameters of the Caps-CB model when the router iteration number gradually increased from two to six, and the results are shown in Table 4. As shown in Table 4, the number of parameters in our model does not change with an increase in the iteration number; however, there is a small decrease in the WER of the model. The WER is the lowest when the number of iterations is two. This is because the dynamic routing algorithm iterates only for parameter updating and optimization and does not expand the number of parameters. As the iteration number increases, the number of parameter updates, matrix multiplications, and complex operations during the training process increase, thus, slowing down the training rate of the capsule network and reducing the convergence degree of the network. Therefore, in practical speech recognitions, it is not necessary to increase the number of the dynamic router iterations more than two.

#### 4.3.3. Influence of the Beam Size

**Figure 9:**
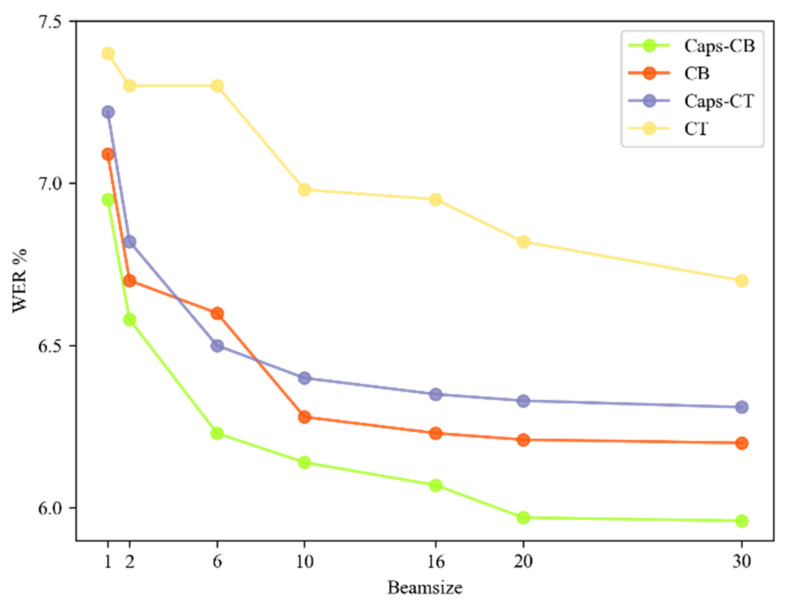
WERs of different models with variable beam sizes.

Because the attention rescoring method was adopted in our decoding process, to explore the model prediction performance, we calculated the WER curves of the Caps-CB, CB, Caps-CT and CT models when different beam sizes were chosen in the CTC search; the results are shown in Figure 9. It can be observed that the recognition performance of all models shows a significant improvement when the value of the beam size increases from 1 to 20, however, the WER curves tends to be flat when the beam size further increases, that is, the recognition performance gain of these models reaches a saturation state. This is because increasing the beam size can improve the diversity of the generated texts, but simultaneously, the similarity between the generated texts becomes higher. When the diversity of samples is not sufficiently high, the recognition accuracy of these model tends to be constant or even decrease. As compared with these models, the WER curve of the Caps-CB model proposed in this study decreased the fastest with increasing beam size, which was due to the fast convergence speed and good fitting effect of Caps-CB, and it did not decrease significantly even when the beam size was equal to 30, while the WER of the CT model decreased the slowest, followed by the Caps-CT and the CB models. This means that the bidirectional transformer can decode in both directions, which fully uses the contextual information and extends the modeling possibilities.

Therefore, when designing a speech-recognition model, it is necessary to select an appropriate beam size.

Finally, we fixed the value of the beam size to be 20, and compared the transcriptions obtained by different models with the same discourse in AISHELL-1. The results are shown in Table 5. All the models were trained using the full dataset, and the converged models were used for prediction. From Table 5, we can observe that the output of Caps-CB is more accurate and grammatically correct, whereas both the LAS and CB models have some error cases, such as the underlined parts in the table. These results prove that the Caps-CB model in this study has better generalization ability and practical performance. It also shows that the capsule network is more effective for extracting deep speech features and semantic logic, and therefore, to further exploit the location information of utterances. There are good results on smaller databases and the performance is able to outperform ordinary CNN layers.

## 5. Conclusions

To address the problems of incomplete feature extraction and limited learning accuracy of existing speech processing models based on a conformer encoder, in this study, a method of using a capsule network to improve the accuracy of feature extraction based on a conformer encoder was studied, and then, an end-to-end speech recognition model was proposed. Our primary contribution is the introduction of the capsule network into the conformer model architecture to improve the speech feature learning accuracy with its powerful feature extraction ability. The second contribution is the simultaneous application of the residual network in the capsule blocks to reduce the training difficulty of the capsule network. Third, we built a series of similar architectures using the CTC/AED model, which significantly improved the training speed and stability, and conducted several comparison experiments on the AISHELL-1 dataset. The experimental results demonstrate that our Caps-CB model achieves a fast convergence speed and higher recognition accuracy. In addition, because our model does not require a language model, the modeling process is simple, and the error analysis results show that it can achieve speech recognition with high precision. Therefore, our model which combines a capsule network and a conformer encoder has the potential for other practical applications related to speech processing because of its high accuracy of speech extraction and learning.

## Figures and Tables

**Table 1:** Comparison of speech recognition models with different encoder and decoders.

Models	WER (%)	Parameter Quantity (M)
CNN × 2 + ResconvLSTM × 8 + DNN + CTC	17.59	-
LAS	13.20	-
CB	6.21	50.0
CT	6.82	47.8
TT	7.50	33.8
TB	6.55	35.8
Caps-TB	6.29	37.7
Caps-TT	7.31	35.5
Caps-CT	6.36	49.7
Caps-CB	5.97	51.9

**Table 2:** Performance comparisons of the recognition models based on the performer encoder.

Model	Enc-Dec-Dm-Head	WER (%)	Parameter Quantity (M)
Caps-PB	12-6-256-4	8.01	36.1
Caps-CB	12-6-256-4	5.97	51.9
PB	12-6-256-4	8.24	34.2
CB	12-6-256-4	6.21	50.0

**Table 3:** Performance improvement after parameter balance and analysis.

	CB	CB_enlarged	Caps-CB
WER (%)	6.21	6.18	5.97
Parameter quantity (M)	50.00	51.95	51.94

**Table 4:** Performance achieved with different router iterations in the capsule network.

	2	3	4	5	6
WER (%)	5.97	6.25	6.23	6.27	6.28
Parameter quantity (M)	51.9	51.9	51.9	51.9	51.9

**Table 5:** Transcription performance in Chinese with different models when the beam size was 20.

Models	Transcription
Truth	这令被贷款的员工们寝食难安
(a) LAS	这令被带款的员工们寝室男安
(b) CB	这令被贷款的员工们请是男安
(c) Caps-CB	这令被贷款的员工们**寝食难安**
Truth	按照扶优扶大扶强的原则
(a) LAS	按照福悠福大幅强的原则
(b) CB	按照富有扶大扶强的原则
(c) Caps-CB	按照**扶优扶大扶强**的原则

## Data Availability

Not applicable.

## Acronyms

AED	Attention-based Encoder-Decoder
Bi-transformer	Bidirectional-Transformer
CapsNet	Capsule Network
CNN	Convolutional Neural Network
CTC	Connectionist Temporal Classification
LAS	Listen, Attend and Spell
LSTM	Long Short-Term Memory
MHSA	Multi-Head Self Attention Module
RNN	Recurrent Neural Network
VGGNet	Visual Geometry Group Network
WERs	Word Error Rates
